# Advances in Portable and Wearable Sensing Systems for Biochemical Monitoring

**DOI:** 10.3390/bios16040182

**Published:** 2026-03-24

**Authors:** Xuexing Zhao, Zetao Chen, Yanli Lu

**Affiliations:** 1 School of Disaster and Emergency Medicine, Tianjin University, Tianjin 300072, China; 2 Wenzhou Safety (Emergency) Institute of Tianjin University, Wenzhou 325026, China

## 1. Introduction

Biological detection technologies play a pivotal role in disease diagnosis and health management. However, traditional centralized testing methods often rely on invasive sampling and bulky laboratory equipment, making it difficult for them to meet the growing demands for real-time monitoring and emergency diagnosis. To bridge this gap, wearable biosensors have emerged as a transformative solution. Characterized by their non-invasive nature, lightweight design, and capability for continuous physiological monitoring, these devices capture biofluids to provide critical data support for precision medicine across diverse scenarios, from maternal health to chronic disease management.

We launched the Special Issue “Advances in Portable and Wearable Sensing Systems for Biochemical Monitoring” to highlight the latest technological evolutions and promote the paradigm shift from “passive treatment” to “active prevention”. This collection aims to address the critical hurdles in translating these technologies from laboratory prototypes to clinical-grade applications, focusing on synergistic innovations in functional materials, structural designs, and system integration.

**Figure 1:**
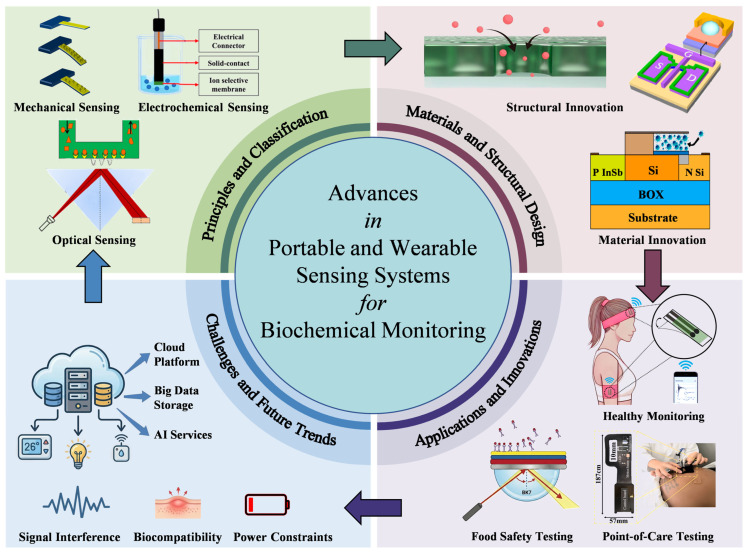
Advances in portable and wearable sensing systems for biochemical monitoring.

This Special Issue successfully collects twelve outstanding contributions, comprising five review articles and seven original research papers. A visual summary of the sensing systems and applications covered in this Special Issue is presented in Figure 1. These works cover a wide spectrum of innovations, ranging from core sensing principles (including optical, electrochemical, and mechanical modalities) to diverse application scenarios such as food safety and sports science. In the following sections, we will briefly introduce these contributions and discuss how they collectively tackle current challenges like signal stability and biocompatibility, while pointing towards future trends in Artificial Intelligence (AI) and Internet of Things (IoT) integration.

The contributions in this Special Issue can be grouped into three distinct technological themes: innovations in electrochemical and mechanical sensing interfaces, advances in non-invasive optical monitoring, and the realization of integrated systems for specific clinical needs.

## 2. Overview of Contributions

### 2.1. Pushing the Limits of Electrochemical and Mechanical Sensing

High sensitivity and miniaturization remain the paramount objectives of bio-interface design. One of the fundamental challenges in field-effect transistor (FET) biosensors is the Debye screening effect, which limits detection in high-ionic-strength fluids. Addressing this, Lee and Cho (Contribution 1) present an emerging Dual-Gate FET (DG-FET) paradigm. By leveraging capacitive coupling, they successfully amplified signals for ultra-low-concentration cortisol detection, overcoming the screening limitations inherent to complex bioenvironments.

Expanding the sensing landscape beyond electrical signals, Muhammad et al. (Contribution 10) provide a critical review of silicon-based biosensors that encompasses mechanical modalities. They highlight how micro-electromechanical systems (MEMS), specifically micro-cantilevers, can translate biomolecular binding events into measurable mechanical deflections or resonant frequency shifts. This label-free mechanical approach complements electrical detection methods like silicon nanowires, offering a versatile toolkit for detecting analytes where charge transfer is limited.

Beyond sensitivity, stability in wearable form factors is crucial. Fathy and Bühlmann (Contribution 9) review the evolution of solid-contact ion-selective electrodes (SC-ISEs), emphasizing the pivotal role of conductive polymers in eliminating the “water layer” effect, thus enabling stable, calibration-free sweat analysis. Furthermore, Li et al. (Contribution 2) demonstrates a novel approach to stress monitoring by combining reverse iontophoresis with magnetic bead-based sensing. Their work effectively balances the need for non-invasive interstitial fluid extraction with the demand for a regenerable sensing interface.

### 2.2. The Renaissance of Optical and Spectroscopic Monitoring

Optical methods offer the unique advantage of being non-contact and often label-free. In the domain of food safety, Hu et al. (Contribution 6) discuss the evolution of surface plasmon resonance (SPR) technology. They illustrate how SPR is transitioning from bulky lab instruments to portable pathogen detectors capable of rapid screening without complex pretreatment.

For physiological monitoring, Hsiao et al. (Contribution 7) introduce a machine learning-enhanced multi-wavelength photoplethysmography (PPG) system. Unlike traditional heart-rate monitors, their device directly estimates oxygen consumption (VO_2_) by analyzing the quasi-DC component of optical signals, significantly reducing motion artifacts during exercise.

Material innovation continues to drive optical sensing forward. Tiwari and Narayan (Contribution 12) investigate the role of polymeric stabilizing agents in gold nanoparticle-mediated Fluorescence Resonance Energy Transfer (FRET) biosensing. Their findings underscore the importance of precise molecular spacing control in maximizing signal-to-noise ratios. Additionally, Wang et al. (Contribution 8) present a mobile, wireless system for skin autofluorescence (SAF) detection. By targeting advanced glycation end products (AGEs), their device provides a non-invasive window into long-term metabolic health and diabetic complications.

### 2.3. Integrated Systems: From Components to Clinical Solutions

Perhaps the most exciting trend in this Special Issue is the integration of these sensing technologies into application-specific devices. Qu et al. (Contribution 3) developed a portable electrochemiluminescence (ECL) device featuring vertically aligned silica mesochannels (VSM). By utilizing the electrostatic enrichment effect of nanopores, they achieved pg/mL-level detection of tumor necrosis factor alpha (TNF-α) in minute volumes of human tears, marking a significant step toward point-of-care ophthalmic diagnostics.

Respiratory health monitoring has also seen innovative form factors. Mestre et al. (Contribution 4) integrated a nanoparticle-based immunoassay directly onto face masks, enabling the non-invasive capture and analysis of exhaled aerosols to evaluate airway inflammation in Chronic Obstructive Pulmonary Disease (COPD) patients. In the critical area of maternal care, Nguyen et al. (Contribution 5) showcased a wireless multimodal sensor capable of monitoring transabdominal placental oxygen saturation, providing vital data for fetal safety that was previously difficult to obtain non-invasively. Finally, Vo and Trinh (Contribution 11) review the advancements in wound healing monitoring, highlighting how wearable sensors are transforming wound care from passive dressings into active diagnostic platforms that monitor pH, temperature, and biomarkers in real time.

## 3. Conclusions and Outlook

The research collected in this Special Issue confirms that wearable biosensors have evolved from isolated components into a robust technological ecosystem. The contributions presented here demonstrate that the barrier to continuous, non-invasive monitoring is being significantly lowered through the synergistic innovation of functional materials (such as VSM and conductive polymers) and structural designs (exemplified by smart masks and micro-cantilevers). These advancements collectively validate that the field is steadily moving away from rigid, invasive clinical tools toward flexible, user-centric interfaces.

However, the transition from laboratory prototypes to commercial reality is not yet complete. As highlighted by several contributions in this issue, significant engineering bottlenecks remain. Challenges such as biofouling at the sensor–tissue interface continue to limit the lifespan of implantable and semi-invasive devices. Furthermore, environmental interference—ranging from skin-tone bias in optical sensors to motion artifacts in mechanical sensing—requires more robust correction algorithms. Beyond performance, achieving batch-to-batch consistency in the mass production of nanomaterial-modified electrodes remains a critical hurdle for industrial scalability.

Looking forward, we believe the next frontier lies in the intelligent fusion of sensing and intervention. Future research is envisioned to concentrate on three transformative pillars. First, the field must move beyond “read-only” devices toward closed-loop therapeutics, where biosensors integrated with stimuli-responsive delivery systems autonomously trigger therapeutic responses. Second, with the introduction of enormous data volumes, AI-driven digital twins will become indispensable, allowing sensors to predict health trajectories rather than merely report current states. Finally, considering the environmental impact of ubiquitous wearables, the development of sustainable electronics using bioresorbable or biodegradable materials will be a critical direction for responsible innovation.

In conclusion, the studies in this Special Issue serve as both a testament to current progress and a blueprint for future exploration. We hope this collection inspires researchers to continue bridging the gap between engineering feasibility and clinical utility, ultimately driving the paradigm shift from “passive treatment” to “active prevention”.

